# Variations in Creatinine Generation Among Patients With Glomerular Disease: Evidence From the NEPTUNE and CureGN Studies

**DOI:** 10.1016/j.xkme.2025.101010

**Published:** 2025-04-17

**Authors:** Shalini S. Ramachandra, Melody Chiang, Michael Arbit, Dorey A. Glenn, Laura H. Mariani, Jarcy Zee

**Affiliations:** 1 Division of Biostatistics, Department of Biostatistics, Epidemiology, and Informatics, University of Pennsylvania Perelman School of Medicine, Philadelphia, PA; 2 Divison of Nephrology, Department of Internal Medicine, University of Michigan, Ann Arbor, MI; 3 UNC Kidney Center, University of North Carolina Chapel Hill, Durham, NC; 4 Children’s Hospital of Philadelphia Research Institute, Philadelphia, PA

**Keywords:** Glomerular disease, creatinine generation, creatinine excretion, creatinine production, serum creatinine, GFR, urine protein, steroid use

## Abstract

**Rationale & Objective:**

Estimation of glomerular filtration rate (GFR) assumes that creatinine generation (crG) is relatively stable. This study identified factors associated with crG variability and its impact on serum creatinine changes (Δ Scr) among patients with glomerular disease.

**Study Design:**

An observational cohort study.

**Setting & Participants:**

Nephrotic Syndrome Study Network and Cure Glomerulonephropathy adult and pediatric participants with at least one crG measurement.

**Predictors:**

Potential predictors of crG levels included age, sex, disease diagnosis, weight status, estimated GFR (eGFR), urine protein, steroid use, and nonsteroid immunosuppressant use. crG change (Δ crG) was then used as an exposure to assess impacts on Δ Scr.

**Outcomes:**

crG levels and Δ Scr.

**Analytical Approach:**

The intraclass correlation coefficient illustrated crG variability within individuals. Multivariable linear mixed models identified factors associated with crG levels. Among those with 2+ crG measurements, multivariable linear mixed models estimated the association between Δ crG and Δ Scr.

**Results:**

Among 4,626 crG measurements from 1,081 participants, there was only moderate correlation between measurements within individuals (intraclass correlation coefficient = 0.517, 95% CI, 0.482-0.548) overall. For pediatric participants, factors significantly associated with crG included age, sex, weight status, and urine protein. Among adults, significant factors were age, sex, disease diagnosis, weight status, eGFR, steroid use, and nonsteroid immunosuppressant use.

**Limitations:**

The 24-hour urine collections may have collection error, measured GFR was unavailable, and edema status was unavailable.

**Conclusions:**

crG was highly dynamic within individuals over time and varied with glomerular disease activity and treatments. The impact of Δ crG on Δ Scr —and subsequently on estimation of kidney function—is potentially large. Accounting for these changes or development of alternative kidney function measures are needed among glomerular disease patients.

## Plain Language Summary

Creatinine generation is often assumed to be stable when using creatinine to estimate kidney function and track kidney function over time, but it can vary with chronic disease. This study showed high variability in creatinine generation within individuals with glomerular disease. Besides age, sex, and weight, important factors that can impact creatinine generation include urine protein in children and kidney function, disease diagnosis, steroid use, and nonsteroid immunosuppressant use in adults. Changes in creatinine generation may also impact changes in the serum creatinine, which would influence estimates of kidney function, but this relationship needs to be studied further. In the meantime, accounting for factors affecting creatinine generation or using alternative estimates of kidney function in patients with glomerular diseases is needed.

Serum concentrations of filtration biomarkers like creatinine are used to estimate glomerular filtration rate (GFR).^1^^,^^2^ However, these equations assume that skeletal muscle creatinine generation is within a normal range and is stable over time. Creatinine generation changes with age-related changes in muscle mass, increasing from infancy through adolescence and decreasing in older age.^3^^,^^4^ Low creatinine generation is also associated with chronic or critical illness, hepatic disease, and long-term corticosteroid use, potentially due to decreases in muscle mass resulting from these states.^5^, ^6^, ^7^ However, little is known about how creatinine generation is impacted by glomerular disease activity or medication use, and it is unclear whether increases in serum creatinine levels in these conditions are due to changes in glomerular filtration or creatinine generation itself. Understanding the relationship between creatinine generation and glomerular disease activity has additional implications for patient outcomes and prognosis, as decreased creatinine generation is associated with increased mortality and other patient outcomes.^8^^,^^9^

In this study, we combined data from 2 multicenter prospective observational cohort studies to examine variability in creatinine generation among patients with glomerular disease. We hypothesized that lower creatinine generation would be associated with higher urine protein and steroid use. We also investigated the potential impact of changes in creatinine generation on changes in serum creatinine to understand implications on estimation of kidney function.

## Methods

### Data Sources and Sample Selection

Data from the Nephrotic Syndrome Study Network (NEPTUNE) and Cure Glomerulonephropathy (CureGN) network were combined for this study. NEPTUNE is a multicenter, observational cohort study in North America enrolling adult and pediatric (Ped) patients at the time of a clinically indicated biopsy with minimal change disease (MCD), focal segmental glomerulosclerosis (FSGS), and membranous nephropathy (MN).^10^ Patients with immunoglobulin A nephropathy (IgAN) and nonbiopsied Ped patients with nephrotic syndrome who were enrolled in NEPTUNE were also included in this study, whereas those with other diagnoses were excluded. NEPTUNE participants enrolled between 2009 and July 2023 were eligible for this study.

CureGN is a multicenter, observational cohort study with Ped and adult patients, with sites across North America, Italy, and Poland.^11^ Participants must have a biopsy-confirmed diagnosis of MCD, FSGS, MN, or IgAN with the diagnostic biopsy within 5 years before enrollment. Some NEPTUNE study participants were also enrolled in CureGN (NEPTUNE transfers) and appear in both datasets. Duplicated data were removed after combining the 2 datasets. CureGN participants enrolled between 2014 and September 2023 were eligible for inclusion.

All participants in NEPTUNE and CureGN provided written informed consent (adults) or assent with parental written consent (children) at study enrollment. Study visits occurred every 4-6 months, during which demographic and clinical variables were collected. As reporting race and ethnicity was mandated by the US National Institutes of Health, consistent with the Inclusion of Women, Minorities, and Children policy, race was self-reported or reported by parents of Ped patients. Study participants with at least one 24-hour timed urine collection that could be used to calculate creatinine generation were eligible for this study. In addition, study participants were required to have a urine protein measured within 30 days, serum creatinine measured within 6 months, and height and weight measured within 1 year of the 24-hour urine collection date. This study was considered not human subjects research and did not require formal review by The Children’s Hospital of Philadelphia institutional review board (IRB #22-020363).

### Calculation of Creatinine Generation

Only outpatient 24-hour timed urine collections were used, and we assumed study participants were in a steady state during the 24-hour collection period to justify the use of 24-hour urine creatinine excretion to represent creatinine generation.^12^, ^13^, ^14^ The following equation was used to calculate creatinine generation (crG) in mg/kg/day from each 24-hour timed urine collection:

$$\text{crG}\;\left(\text{mg}/\text{kg}/\text{day}\right)=\frac{\text{creatinine}\;\text{concentration}\;\left(\text{mg}/\text{dL}\right)\;\times \;\text{urine}\;\text{volume}\;\left(\text{mL}\right)}{100\;\times \;\text{number}\;\text{of}\;\text{days}\;\times \;\text{weight}\;\left(\text{kg}\right)}\text{.}$$

Creatinine concentrations greater than 600 mg/dL and urine volumes greater than 7,000 mL were considered errors and removed. Number of days refers to the urine collection timing (converted to days) to account for any urine collections that were not exactly 24 hours long. Any collections with missing timings were assumed to be exactly 1 day long. The closest weight measurement within 1 year was used. Creatinine generation was considered missing if creatinine concentration or volume was missing (or removed) or if there was no weight measurement available within 1 year before or after the urine collection date. To further mitigate the potential for data entry errors to cause extreme values, crG values below the 5th percentile and above the 95th percentile across all observed values in the study sample were excluded from analysis. Non-weight-normalized crG (g/day) was also calculated for supplementary analyses.

### Other Variable Definitions

The nearest urine protein, serum creatinine, height, and weight were matched to each creatinine generation measurement. Centrally measured values for urine protein and serum creatinine were used whenever available, but otherwise measurements reported by study sites were used. Urine protein was represented by urine protein creatinine ratio (UPCR), calculated from the same 24-hour timed urine collection whenever available, or otherwise by the closest first morning void or random spot urine collection. If UPCR was not available, urine albumin creatinine ratio (UACR) was used. Disease activity categories were defined as: remission (UPCR or UACR < 0.3), partial remission (0.3 ≤ UPCR or UACR < 1.5), active (1.5 ≤ UPCR or UACR < 3), and nephrotic (UPCR or UACR ≥ 3).

The eGFR was calculated by the race-agnostic Chronic Kidney Disease Epidemiology Collaboration 2021 (CKD-Epi) formula for participants 25+ years of age and the under 25 (U25) formula for participants <25 at the time of serum creatinine measurement.^2^^,^^15^ The CKD stages were defined by eGFR alone.^16^

Body mass index (BMI) was calculated based on the closest height and weight measurements to each creatinine generation value. The BMI percentiles (BMIPct) for Ped patients were calculated based on 2000 Centers for Disease and Control (CDC) growth charts (using the cdcanthro function in the cdcanthro R package). Weight statuses were defined as: underweight (BMI: < 18.5 or BMIPct: < 5 for Ped), normal weight (BMI: 18.5-<25 or BMIPct: 5-<85 for Ped), overweight (BMI: 25-<30 or BMIPct: 85-<95 for Ped), and obese (BMI: ≥30 or BMIPct: ≥95 for Ped).

Binary steroid and non-steroid immunosuppressant (IST) use indicators at the time of each creatinine generation measurement were determined based on medication start and stop dates in the NEPTUNE and CureGN databases. Non-steroid ISTs included calcineurin inhibitors, mycophenolate mofetil, cyclophosphamide, and rituximab, among others.

### Statistical Analysis

Descriptive statistics were reported by study and overall to summarize demographics, characteristics of patients at study enrollment, and clinical characteristics at the time of creatinine generation measurements. The intraclass correlation coefficient (ICC), measuring variability within individuals for repeated observations, was calculated overall and within subgroups. The ICC was calculated as a ratio of between-individual variability and the sum of between-individual and within-individual variability based on a random effects model (and is therefore bounded by zero); thus, individuals with only 1 creatinine generation measurement contributed to estimates of between-individual variability and those with 2+ measurements contributed to estimates of between-individual and within-individual variability. Parametrically bootstrapped 95% confidence intervals were calculated and reported for each ICC using the bootMer function in the lme4 R package with 1,000 iterations. The overall coefficient of variation was also calculated within subjects.

Multivariable linear mixed models were used to identify factors associated with creatinine generation levels, with random intercepts for each study participant to account for repeated observations within individuals. Covariates included age, sex, disease diagnosis, weight status, eGFR (as a proxy for measured kidney function), urine protein, steroid use, and non-steroid IST use. Urine protein was log-2 transformed to allow for interpretation of coefficients for a doubling of urine protein. Pearson residual plots and random intercept quantile-quantile plots were used to assess model assumptions. Interactions between steroid use and disease diagnosis category were included in models. In supplementary analyses, these models were repeated using non-weight-normalized creatinine generation.

To quantify the degree to which variability in creatinine generation may mislead estimates of kidney function based on serum creatinine, we analyzed the adjusted association between change in creatinine generation and change in serum creatinine. Among study participants with 2+ creatinine generation measurements, the change in creatinine generation and change in serum creatinine was calculated between each pair of adjacent measurements. For these analyses, serum creatinine was required to be within 7 days of the creatinine generation measurement. Four-category variables were created to represent changes in disease activity and steroid use (eg, in remission during the first observation of the pair (T1), second observation of the pair (T2), both, or neither). Age and weight status were assessed during the first observation (T1) of each pair, and sex and disease diagnosis remained constant. Multivariable linear mixed models were then used to estimate the adjusted association between changes in creatinine generation and changes in serum creatinine. The number of months between serum creatinine measurements (T1 to T2) was included as an additional adjustment covariate, whereas eGFR was excluded from these models since it would be derived from the serum creatinine outcome. Interactions between changes in creatinine generation and 3 factors were considered: disease diagnosis, steroid use, and changes in disease activity. This analysis was also repeated using non-weight-normalized creatinine generation.

The association between changes in creatinine generation and changes in serum creatinine is likely confounded by measured GFR (mGFR), but mGFR was not available.^17^ Therefore, we used a simulation study and generated synthetic GFR values based on the assumptions that mGFR is positively associated with creatinine generation^6^^,^^18^^,^^19^ and negatively associated with serum creatinine. The GFR was simulated using a linear model and varying coefficient magnitudes of creatinine generation change and serum creatinine change. We then considered how linear mixed model results of the adjusted association between changes in creatinine generation and changes in serum creatinine would differ after additionally controlling for simulated GFR. For each set of simulation parameters, we repeated the simulation 500 times and averaged results across simulation repetitions.

Models were fitted among the overall sample and within subgroups of Ped versus adult age at first creatinine generation measurement. All analyses were conducted using R version 4.2.1 (R Core Team).

## Results

**Figure 1:**
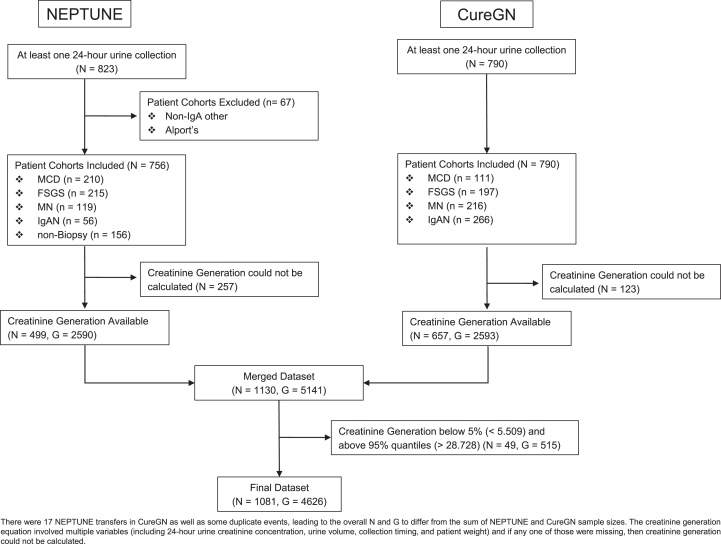
Sample selection flow diagram.

A total of 1,081 study participants contributed 4,626 unique creatinine generation measurements (Fig 1). There were 488 study participants from NEPTUNE and 610 study participants from CureGN, including 17 NEPTUNE transfers in both datasets, so the overall sample size is not the sum from the 2 studies (Table S1). Over half of all participants were male (59%), 68% reported White/Caucasian race, and 15% reported Black/African American race (Table 1). At study enrollment, the mean ± SD age was 37 ± 21, median (IQR) eGFR was 80 (53-100), and median (IQR) urine protein was 1.9 (0.55-4.7). At the time of creatinine generation measurements, the median (IQR) eGFR was 78 (50-104) and urine protein was 0.8 (0.18-2.5) (Table 2). Most participants were not on steroids (75%) or nonsteroid ISTs (69%). The median (IQR) absolute value of time between serum creatinine and creatinine generation measurements was 0.0 (0.0-1.0) days. The median (IQR) time between creatinine generation measurements among those with 2+ measurements was 4.87 (2.76-7.76) months.

**Table 1:** Demographics and clinical characteristics at study enrollment, by age and overall

	Pediatric	Adult	Overall
N	289	792	1,081
Age (y), mean ± SD	10 ± 4.8	39 ± 20	37 ± 21
Sex			
Male	177 (61%)	464 (59%)	641 (59%)
Female	112 (39%)	328 (41%)	440 (41%)
Race			
Asian/Asian American	11 (4%)	111 (14%)	122 (11%)
Black/African American	51 (18%)	108 (14%)	159 (15%)
Multiracial	13 (4%)	19 (2%)	32 (3%)
Native American/Alaskan native/First nations	0 (0%)	2 (< 1%)	2 (< 1%)
Native Hawaiian/Other Pacific Islander	0 (0%)	4 (1%)	4 (< 1%)
White/Caucasian	202 (70%)	533 (67%)	735 (68%)
Unknown	12 (4%)	15 (2%)	27 (2%)
Ethnicity			
Hispanic or Latino	45 (16%)	112 (14%)	157 (15%)
Not Hispanic or Latino	243 (84%)	679 (86%)	922 (85%)
Unknown	1 (< 1%)	1 (< 1%)	2 (< 1%)
Disease diagnosis			
MCD	106 (37%)	103 (13%)	209 (19%)
FSGS	68 (24%)	246 (31%)	314 (29%)
MN	14 (5%)	260 (33%)	274 (25%)
IgAN	66 (23%)	183 (23%)	249 (23%)
Non-biopsy	35 (12%)		35 (3%)
eGFR (mL/min/1.73m^2^),^a^ median (IQR)	93 (77-110)	71 (47-96)	80 (53-100)
Urine protein (g/g),^b^ median (IQR)	0.69 (0.16-5)	2.3 (0.83-4.7)	1.9 (0.55-4.7)
Creatinine generation (mg/kg/day), median (IQR)	16 (12-20)	16 (13-20)	16 (13-20)
Non-weight-normalized creatinine generation (g/day), median (IQR)	0.66 (0.37-1.10)	1.36 (0.98-1.77)	1.2 (0.77-1.62)

Abbreviations: eGFR, estimated glomerular filtration rate; FSGS, focal segmental glomerulosclerosis; IgAN, immunoglobulin A nephropathy; MCD, minimal change disease; MN, membranous nephropathy.

a Missing for 60 individuals overall: 16 among pediatric and 44 among adults.

b Missing for 64 individuals overall: 11 among pediatric and 53 among adults.

**Table 2:** Clinical Characteristics During crG Measurements

	Pediatric		Adult		Overall	
	# crG	Median (IQR) or %	# crG	Median (IQR) or %	# crG	Median (IQR) or %
Age (y)	1,098	11 (7-15)	3,528	50 (37-62)	4,626	42 (21-59)
Weight status^a^						
Underweight	472	43%	36	1%	508	11%
Normal weight	309	28%	785	23%	1,094	24%
Overweight	131	12%	1267	37%	1,398	31%
Obese	181	17%	1362	39%	1,543	34%
Serum creatinine (mg/dL)	1,098	0.53 (0.41-0.75)	3,528	1.2 (0.88-1.7)	4,626	1 (0.72-1.5)
eGFR (mL/min/1.73m^2^)^b^	1,098	103 (84-121)	3,526	67 (44-95)	4,624	78 (50-104)
CKD Stage^b^						
Normal/high GFR	744	67%	1,044	30%	1,777	38%
Mild CKD	274	25%	974	28%	1,248	27%
Moderate CKD (stage 3A)	39	4%	609	17%	648	14%
Moderate CKD (stage 3B)	28	3%	515	15%	543	12%
Severe CKD	17	2%	333	9%	350	8%
End stage CKD	7	1%	51	1%	58	1%
Urine protein (g/g)	1,098	0.27 (0.10-1.4)	3,528	1 (0.28-2.7)	4,626	0.8 (0.18-2.5)
Disease activity category						
Nephrotic	165	15%	768	22%	933	20%
Active	105	10%	639	18%	744	16%
Partial remission	267	24%	1,200	34%	1,467	32%
Remission	561	51%	921	26%	1,482	32%
Steroid Use						
Not on steroids	658	60%	2,832	80%	3,490	75%
On steroids	440	40%	696	20%	1136	25%
Non-steroid IST Use						
Not on non-steroid ISTs	551	50%	2,647	75%	3,198	69%
On non-steroid ISTs	547	50%	881	25%	1,428	31%
Creatinine generation (mg/kg/day)	1,098	17 (13-21)	3,528	16 (12-19)	4,626	16 (12-20)
Non-weight-normalized creatinine generation (g/day)	1,098	0.71 (0.42-1.07)	3,528	1.29 (0.95-1.70)	4,626	1.17 (0.79-1.59)

Abbreviations: CKD, chronic kidney disease; crG, creatinine generation; eGFR, estimated glomerular filtration rate; IST, immunosuppressant.

a Missing for 83 crG measurements overall; 5 among pediatric and 78 among adults.

b Missing for 2 crG measurements overall, both among adults.

### Variability of Creatinine Generation Within Individuals

**Figure 2:**
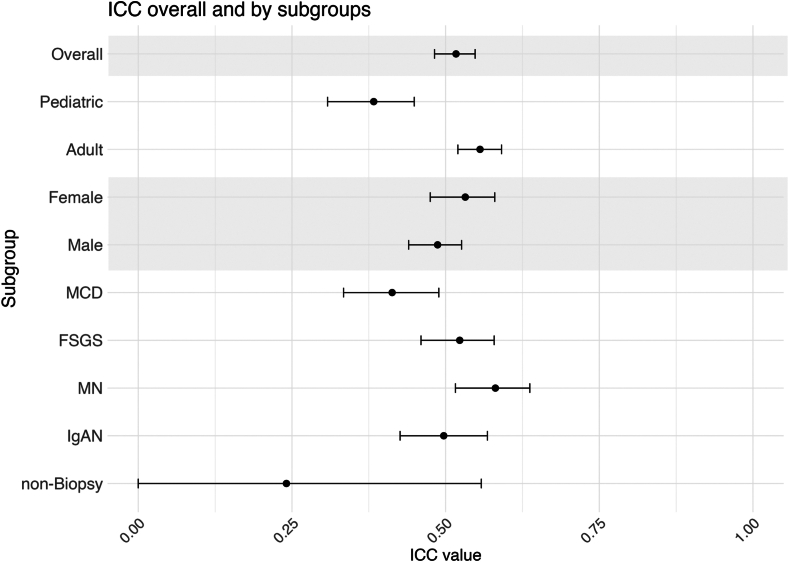
Intraclass correlation coefficient (ICC) by subgroup (age, sex, and disease diagnosis categories) and overall, with 95% confidence interval bars.

The ICC of creatinine generation measurements was 0.517 (95% CI, 0.482-0.548) overall (Fig 2), indicating only moderate commonality between measurements within an individual. Among the 720 study participants (67% of 1,081) with 2+ creatinine generation measurements, the median coefficient of variation was 0.205 (0.123-0.303), indicating a standard deviation of 20.5% of the mean. Among the 563 study participants (52% of 1081) with 3+ creatinine generation measurements, the median coefficient of variation increases slightly to 0.221 (0.144-0.305).

Among Ped study participants, the ICC was 0.383 (95% CI: 0.308-0.449), whereas the ICC for adults was 0.556 (95% CI, 0.520-0.591) (Fig 2). Stratifying by sex, the ICC was 0.532 (95% CI, 0.475-0.580) for females and 0.487 (95% CI, 0.440-0.526) for males. Across disease diagnoses, the ICC was 0.413 (95% CI, 0.334-0.489) for those with MCD, 0.523 (95% CI, 0.460-0.579) for FSGS, 0.581 (95% CI, 0.516-0.637) for MN, 0.497 (95% CI, 0.426 0.568) for IgAN, and 0.241 (95% CI, 0.000-0.558) for nonbiopsied study participants.

### Factors Associated with Creatinine Generation

After adjustment for other covariates, factors significantly associated with creatinine generation included age, sex, disease diagnosis, weight status, eGFR, steroid use, and non-steroid IST use. However, some of these associations differed among Ped versus adult study participants (Table 3). Older age was significantly associated with higher creatinine generation among Ped study participants (0.69 mg/kg/day per 5 years, 95% CI, 0.23-1.16) and lower creatinine generation among adults (−0.25 per 5 years, 95% CI, −0.35, to −0.15). A significant interaction was found between disease diagnosis and steroid use for adults, but it was neither statistically significant among Ped study participants nor when creatinine generation was not weight-normalized in adults (Table S2). Among adults on steroids, IgAN study participants had 1.34 mg/kg/day higher creatinine generation compared to MCD; and among adults not on steroids, MN and IgAN study participants had 1.52 and 2.62 mg/kg/day higher creatinine generation when compared with MCD, respectively. Children without biopsies and either on or not on steroids had 2.07 or 2.82 mg/kg/day higher creatinine generation compared with MCD, respectively. Within MN and IgAN, adults on steroids had 0.89 and 1.38 mg/kg/day lower creatinine generation compared with those not on steroids, respectively, whereas steroid use was not significantly associated with creatinine generation among adults with MCD and FSGS.

**Table 3:** Factors Associated With Creatinine Generation Among Pediatric and Adult Subgroups

	Pediatric		Adult	
	Estimate (95% CI)	*P*	Estimate (95% CI)	*P*
Age (per 5 y)	0.69 (0.23 to 1.16)	0.004	−0.25 (−0.35 to −0.15)	< 0.001
Female sex	−1.26 (−2.13 to −0.40)	0.006	−2.65 (−3.24 to −2.05)	< 0.001
Weight status (ref: normal weight)		< 0.001		< 0.001
Underweight	−4.52 (−5.66 to −3.43)		1.26 (−0.33 to 2.87)	
Overweight	−2.10 (−3.32 to −0.89)		−0.81 (−1.34 to −0.29)	
Obese	−2.98 (−4.21 to −1.80)		−1.94 (−2.53 to −1.35)	
eGFR (per 5 mL/min/1.73m^2^)	0.03 (−0.03 to 0.08)	0.32	0.08 (0.04 to 0.12)	< 0.001
Urine protein (per doubling)	−0.17 (−0.28 to −0.05)	0.005	0.02 (−0.05 to 0.09)	0.51
Non-steroid IST Use	0.16 (−0.55 to 0.90)	0.67	0.76 (0.36 to 1.18)	< 0.001
Disease diagnosis × steroid use		0.97		0.03
On steroids (ref: MCD)				
FSGS	−0.73 (−2.30 to 0.84)		0.71 (−0.56 to 1.98)	
MN	0.02 (−2.65 to 2.69)		0.74 (−0.57 to 2.05)	
IgAN	−0.31 (−2.05 to 1.43)		1.34 (0.01 to 2.67)	
Non-biopsy	2.07 (0.13 to 4.01)			
Not on Steroids (ref: MCD)				
FSGS	−0.33 (−1.67 to 1.01)		0.50 (−0.56 to 1.56)	
MN	−0.18 (−2.61 to 2.25)		1.52 (0.46 to 2.58)	
IgAN	−0.10 (−1.56 to 1.35)		2.62 (1.51 to 3.72)	
Non-biopsy	2.82 (0.27 to 5.37)			
Steroid Use among MCD	0.38 (−0.52 to 1.28)		−0.10 (−1.09 to 0.89)	
Steroid use among FSGS	−0.03 (−1.44 to 1.38)		0.11 (−0.64 to 0.86)	
Steroid use among MN	0.57 (−1.94 to 3.08)		−0.89 (−1.65 to −0.13)	
Steroid use among IgAN	0.17 (−1.34 to 1.68)		−1.38 (−2.18 to −0.58)	
Steroid use among non-biopsy	−0.37 (−3.07 to 2.33)			

*Note:* Estimates are from linear mixed models with creatinine generation as the outcome and random intercepts for each study participant to account for repeated measures within individuals. The multivariable model among pediatric study participants includes 1,094 creatinine generation measurements from 289 study participants. The multivariable model among adult study participants includes 3,445 creatinine generation measurements from 783 study participation.

Abbreviations: eGFR, estimated glomerular filtration rate; FSGS, focal segmental glomerulosclerosis; IgAN, immunoglobulin A nephropathy; IST, immunosuppressant; MCD, minimal change disease; MN, membranous nephropathy.

Weight status had an inverted U-shaped association with creatinine generation (per kg body weight) among children (Table 3). In contrast, creatinine generation (per kg body weight) decreased monotonically with increasing weight status among adults (Table 3) and nonweight-normalized creatinine generation increased monotonically with increasing weight status among adults (Table S2). The eGFR and nonsteroid IST use were not significantly associated with creatinine generation (mg/kg/day) among children, whereas higher eGFR and non-steroid IST use were associated with higher creatinine generation among adults. Higher urine protein was associated with lower creatinine generation among children (−0.17 mg/kg/day per doubling) but was not significantly associated with creatinine generation in adults.

### Associations Between Changes in Creatinine Generation and Changes in Serum Creatinine

There were 635 study participants with 2+ creatinine generation measurements with corresponding serum creatinine available, resulting in 2,930 pairs of measurements to calculate changes. After controlling for other covariates, changes in creatinine generation were not significantly associated with changes in serum creatinine, overall or within age subgroups (Table 4 first row; Table S3). Similar results were found for changes in non-weight-normalized creatinine generation among adults (Table S4). Interactions between changes in creatinine generation with disease diagnosis, with steroid use, and with disease activity were not statistically significant and therefore removed from models. The association between changes in creatinine generation and changes in serum creatinine is likely confounded by mGFR, which was unavailable. Table 4 shows these association estimates after controlling for simulated GFR. Results demonstrate that with a simulated GFR that is moderately correlated with creatinine generation change (ρ = 0.512) and strongly correlated with serum creatinine change (ρ = −0.835), every 10 mg/kg/day increase in the change in creatinine generation would be associated with a 0.45 mg/dL increase in the change in serum creatinine. This would correspond to a 36.21 or 27.10 mL/min/1.73m^2^ decrease in eGFR for a 35-year-old adult male or female with starting serum creatinine level of 1 mg/dL, respectively, using the CKD-Epi eGFR equations (Table S5).^2^ Similarly, this would correspond to a 14.99 or 18.32 mL/min/1.73m^2^ decrease in eGFR for an 8-year-old male or female at 50^th^ percentile height, respectively, using the U25 equations (Table S5).^15^^,^^20^

**Table 4:** Associations Between Changes in crG and Changes in Scr Before (row 1) and After Adjustment for Simulated GFR

Linear Model Coefficients		Correlations with Simulated GFR		Association between Δ crG (per 10 mg/kg/day) and Δ Scr	
$\beta_{1}$	$\beta_{2}$	Δ crG	Δ Scr	Estimate (95% CI)	*P*
--	--	--	--	0.004 (−0.02 to 0.03)	0.78
0.1	−10	0.116	−0.967	0.09 (0.09-0.11)	< 0.001
0.3	−10	0.330	−0.919	0.27 (0.26-0.28)	< 0.001
0.1	−1	0.403	−0.358	0.08 (0.05-0.11)	< 0.001
0.3	−3	0.705	−0.545	0.45 (0.42-0.48)	< 0.001
0.5	−10	0.512	−0.835	0.45 (0.44-0.46)	< 0.001
1	−20	0.517	−0.849	0.49 (0.48-0.49)	< 0.001
0.5	−5	0.744	−0.601	0.69 (0.66-0.71)	< 0.001
1	−1	0.979	−0.078	0.78 (0.68-0.88)	< 0.001
1	−10	0.766	−0.624	0.90 (0.89-0.92)	< 0.001
2	−20	0.771	−0.631	0.97 (0.97-0.98)	< 0.001
1	−5	0.912	−0.368	1.37 (1.33-1.41)	< 0.001
1	−3	0.953	−0.237	1.47 (1.41-1.54)	< 0.001
2	−10	0.921	−0.376	1.80 (1.77-1.82)	< 0.001

*Note:* GFR was simulated 500 times from the linear model $\text{simulatedGFR}=75+\beta_{1}\Delta \text{crG}+\beta_{2}\Delta \text{Scr}+\varepsilon$, where ΔcrG represents change in creatinine generation per pair of observations in an individual, ΔScr represents change in serum creatinine per pair of observations in an individual, and ε represents a random error term drawn from a standard normal distribution.

Correlations are calculated using Pearson’s correlation coefficient. Five hundred simulations to generated simulated GFR were run and fit to a linear mixed model each time. Association estimates and *P*-values are from a linear mixed model with change in serum creatinine as the outcome and change in creatinine generation as the primary exposure, with random intercepts for each study participant to account for repeated measures within individuals. Models were adjusted for simulated GFR, age, sex, weight status, disease diagnosis, remission status, steroid use, and time in between pairs of measurements. Estimates of the adjusted association between ΔcrG and ΔScr were extracted, and the average of the point estimate and standard error were taken to compute the 95% CI and *P*-value above.

Abbreviations: crG, creatinine generation; GFR, glomerular filtration rate; Scr, serum creatinine.

## Discussion

We found high variability in creatinine generation measurements within patients with glomerular disease, especially among children. Among children, we observed significant negative associations between all weight status categories and creatinine generation compared with normal weight and negative associations between urine protein and creatinine generation. Among adults, creatinine generation (per kg body weight) decreased with increasing weight, and steroid use was significantly negatively associated with creatinine generation only within MN and IgAN subgroups.

Variation in creatinine generation has been investigated in different contexts previously. In healthy individuals, creatinine generation is expected to vary by as little as 4-8%, with higher values among those engaging in strenuous exercise or high animal protein diets.^13^^,^^14^^,^^21^^,^^22^ In contrast, up to 4-fold changes were observed among a small number of patients in an intensive care unit setting.^23^ In individuals with diabetes, variation ranges from 15%-18% with correlations 0.64-0.65,^13^^,^^24^ and creatinine generation decreases by an average of 16 mg/day per year in advanced CKD.^25^ Our study indicates that glomerular disease patients experience similar or even more variability than these chronic disease populations, despite the majority not having advanced CKD. For children in particular, creatinine generation variability was even higher than that of adults and associated with proteinuria, implying that the variability cannot be fully explained by growth (i.e., age, sex, and weight status).

Given the known catabolic effects of corticosteroids, our finding of lower creatinine generation in adult MN and IgAN study participants taking steroids at the time of measurement was not surprising. Interestingly, we did not observe this association in Ped study participants, which may be due to differences in steroid duration and dosing, biologic differences in metabolism or overall smaller muscle mass.^26^ Relatedly, adults with IgAN (whether or not on steroids), MN patients not on steroids, and non-biopsied children with nephrotic syndrome had the highest creatinine generation compared with other disease diagnoses, which may also be due to less cumulative corticosteroid exposure. Future studies on the impacts of cumulative steroid exposure and steroid doses on creatinine generation would help to confirm these hypotheses.

A previous study from the Chronic Renal Insufficiency Cohort (CRIC) found that higher urine creatinine excretion in mg/day was associated with higher BMI, aligning with our results using nonweight-normalized creatinine generation (Table S2).^27^ Since our primary measure of creatinine generation was normalized per kg of body weight and we observed the opposite relationship in adults, this indicates that total creatinine generation increases with increasing weight but at a lower rate than the rate of weight increase. In a glomerular disease population, this may be because weight gain can often reflect edema rather than increases in muscle mass. In contrast, we found an inverted U-shaped relationship between weight status and creatinine generation in Ped study participants, with children of normal weight having the highest creatinine generation. This implies that the rate of decrease in total creatinine generation outpaces the rate of decreasing weight in children.

Given the large within-person variability in creatinine generation, it would be crucial to understand how changes in creatinine generation affect serum creatinine to understand the impacts on kidney function estimation in the clinical setting. Although we did not find evidence of an association between changes in creatinine generation and changes in serum creatinine, this association was likely confounded by levels of mGFR, as observed in the simulation results. While the true magnitude of the effect is still unclear due to the unknown amount of confounding, several of our simulation results demonstrate that a potentially large change in serum creatinine can be fully explained by a change in creatinine generation with no change in kidney function.

A limitation of our study is the possibility of inaccurately collected 24-hour urine collections. This could contribute to an overestimate of variability in creatinine generation that does not reflect true biologic variability. We have mitigated this concern by removing the most extreme 10% of measurements in our sample. Among the remaining measurements, the expectation is that the user collection error is distributed similarly across subgroups of interest. Future studies with 24-hour urine collections in healthy populations could help elucidate how much variability in creatinine generation is due to user collection error vs. biological changes. In addition, bioimpedance analysis was unavailable to examine body composition. A cystatin C-based eGFR measurement could not be used due to the unavailability of cystatin C measurements in NEPTUNE data and insufficient number of cystatin C measurements within 6 months of urine collection in CureGN data. Furthermore, mGFR being unavailable in the study inhibits clear conclusions on the effects of creatinine generation change on serum creatinine change. Although our simulation results partially address this issue, ideal data would include mGFR. Finally, the impact of weight may be influenced by edema, which was not available for analysis.

Despite these limitations, this study represents the first evaluation of variability in creatinine generation among patients with glomerular disease. The combination of NEPTUNE and CureGN data allowed for analysis of a large sample of creatinine generation measurements among both children and adults. Our study illustrates the importance of considering creatinine generation when using serum creatinine to estimate kidney function. The lack of stability of creatinine generation also emphasizes the continued need for alternative markers of kidney function.^1^

## Supplementary Material

Supplementary File Item S1-S3; Table S1-S5.

## References

[1] Delgado C., Baweja M., Crews D.C. (2022). A unifying approach for GFR estimation: recommendations of the NKF-ASN task force on reassessing the inclusion of race in diagnosing kidney disease. Am J Kidney Dis.

[2] Inker L.A., Eneanya N.D., Coresh J. (2021). New creatinine- and cystatin C–based equations to estimate GFR without race. N Engl J Med.

[3] Mian A.N., Schwartz G.J. (2017). Measurement and estimation of glomerular filtration rate in children. Adv Chronic Kidney Dis.

[4] Shlipak M.G., Katz R., Kestenbaum B. (2009). Rate of kidney function decline in older adults: a comparison using creatinine and cystatin C. Am J Nephrol.

[5] Oterdoom L.H., Van Ree R.M., de Vries A.P.J. (2008). Urinary creatinine excretion reflecting muscle mass is a predictor of mortality and graft loss in renal transplant recipients. Transplantation.

[6] Wilson F.P., Sheehan J.M., Mariani L.H., Berns J.S. (2012). Creatinine generation is reduced in patients requiring continuous venovenous hemodialysis and independently predicts mortality. Nephrol Dial Transplant.

[7] Hoste E.A.J., Damen J., Vanholder R.C. (2005). Assessment of renal function in recently admitted critically ill patients with normal serum creatinine. Nephrol Dial Transplant.

[8] Park J., Mehrotra R., Rhee C.M. (2013). Serum creatinine level, a surrogate of muscle mass, predicts mortality in peritoneal dialysis patients. Nephrol Dial Transplant.

[9] Stads S., Schilder L., Nurmohamed S.A., Cozzolino M. (2018). PLOS One.

[10] Gadegbeku C.A., Gipson D.S., Holzman L.B. (2013). Design of the Nephrotic Syndrome Study Network (NEPTUNE) to evaluate primary glomerular nephropathy by a multidisciplinary approach. Kidney Int.

[11] Mariani L.H., Bomback A.S., Canetta P.A. (2019). CureGN study rationale, design, and methods: establishing a large prospective observational study of glomerular disease. Am J Kidney Dis.

[12] Nielsen P.K., Ladefoged J., Olgaard K. (1994). Lean body mass by Dual Energy X-ray Absorptiometry (DEXA) and by urine and dialysate creatinine recovery in CAPD and pre-dialysis patients compared to normal subjects. Adv Perit Dial.

[13] Kalantari K., Bolton W.K. (2013). A good reason to measure 24-hour urine creatinine excretion, but not to assess kidney function. Clin J Am Soc Nephrol.

[14] Heymsfield S.B., Arteaga C., McManus C., Smith J., Moffitt S. (1983). Measurement of muscle mass in humans: validity of the 24-hour urinary creatinine method. Am J Clin Nutr.

[15] Pierce C.B., Muñoz A., Ng D.K., Warady B.A., Furth S.L., Schwartz G.J. (2021). Age- and sex-dependent clinical equations to estimate glomerular filtration rates in children and young adults with chronic kidney disease. Kidney Int.

[16] Improving Global Outcomes (KDIGO) CKD Work Group (2024). KDIGO 2024 Clinical Practice Guideline for the Evaluation and Management of Chronic Kidney Disease. Kidney Int.

[17] Wilson F.P., Xie D., Anderson A.H. (2014). Urinary creatinine excretion, bioelectrical impedance analysis, and clinical outcomes in patients with CKD: the CRIC study. Clin J Am Soc Nephrol.

[18] Heimbürger O., Stenvinkel P., Bárány P. (2012). The enigma of decreased creatinine generation in acute kidney injury. Nephrol Dial Transplant.

[19] Tynkevich E., Flamant M., Haymann J.P. (2014). Decrease in urinary creatinine excretion in early stage chronic kidney disease. PLOS One.

[20] Centers for Disease Control and Prevention (CDC) (2000). Growth Charts - Clinical Growth Charts. https://www.cdc.gov/growthcharts/cdc-charts.htm.

[21] Forbes G.B., Bruining G.J. (1976). Urinary creatinine excretion and lean body mass. Am J Clin Nutr.

[22] Sawant P.D., Kumar S.A., Wankhede S., Rao D.D. (2018). Creatinine as a normalization factor to estimate the representativeness of urine sample - Intra-subject and inter-subject variability studies. Appl Radiat Isot.

[23] Waikar S.S., Sabbisetti V.S., Bonventre J.V. (2010). Normalization of urinary biomarkers to creatinine during changes in glomerular filtration rate. Kidney Int.

[24] Jacobi D., Lavigne C., Halimi J.M. (2008). Variability in creatinine excretion in adult diabetic, overweight men and women: consequences on creatinine-based classification of renal disease. Diabetes Res Clin Pract.

[25] Di Micco L., Quinn R.R., Ronksley P.E. (2013). Urine creatinine excretion and clinical outcomes in CKD. Clin J Am Soc Nephrol CJASN.

[26] Glenn D.A., Andrews C., Liu Q. (2024). Glucocorticoid Exposure and Infection in Children and Adults With Glomerular Disease: Findings From the Cure Glomerulonephropathy Study. Am J Kidney Dis.

[27] Fotheringham J., Weatherley N., Kawar B., Fogarty D.G., Ellam T. (2014). The body composition and excretory burden of lean, obese, and severely obese individuals has implications for the assessment of chronic kidney disease. Kidney Int.

