# Supplementary material for: Variations in Creatinine Generation Among Patients With Glomerular Disease: Evidence From the NEPTUNE and CureGN Studies
Source: Kidney Med. 2025 Apr 17;7(6):101010. doi: 10.1016/j.xkme.2025.101010 (PMC12152656; doi:10.1016/j.xkme.2025.101010)
Supplement: Supplementary File — Item S1-S3; Table S1-S5. [file mmc1.docx]

**Supplemental Materials**

Table S1. Demographics and clinical characteristics at study enrollment, by study and overall

|  | **NEPTUNE** | **CureGN** | **Overall** |
| --- | --- | --- | --- |
| N | 488 | 610 | 1081^†^ |
| Age (years), mean (SD) | 34 (22) | 39 (20) | 37 (21) |
| Sex  Male  Female | 298 (61%)  190 (39%) | 356 (58%)  254 (42%) | 641 (59%)  440 (41%) |
| Race  Asian / Asian American  Black / African American  Multi-Racial  Native American / Alaskan Native / First Nations  Native Hawaiian / Other Pacific Islander  White/Caucasian  Unknown | 55 (11%)  104 (21%)  21 (4%)  0 (0%)  1 (< 1%)  293 (60%)  14 (3%) | 70 (11%)  56 (9%)  12 (2%)  2 (< 1%)  3 (< 1%)  454 (74%)  13 (2%) | 122 (11%)  159 (15%)  32 (3%)  2 (< 1%)  4 (< 1%)  735 (68%)  27 (2%) |
| Ethnicity  Hispanic or Latino  Not Hispanic or Latino  Unknown | 100 (20%)  386 (79%)  2 (< 1%) | 58 (10%)  552 (90%) | 157 (15%)  922 (85%)  2 (< 1%) |
| Disease Diagnosis  MCD  FSGS  MN  IgAN  Non-Biopsy | 133 (27%)  167 (34%)  101 (21%)  52 (11%)  35 (7%) | 77 (13%)  149 (24%)  178 (29%)  206 (34%) | 209 (19%)  314 (29%)  274 (25%)  249 (23%)  35 (3%) |
| eGFR (mL/min/1.73m^2^),^*^ median (IQR) | 80 (57, 101) | 78 (51, 100) | 80 (53, 100) |
| Urine protein (g/g),^**^ median (IQR) | 2.4 (0.68, 5.3) | 1.6 (0.54, 3.8) | 1.9 (0.55, 4.7) |

^*^eGFR was missing for N=1 in NEPTUNE, N=59 in CureGN, and N=60 overall

^**^Urine protein was missing for N=5 in NEPTUNE, N=59 CureGN, and N=64 overall

^†^There were N=17 NEPTUNE transfers (included in all three columns), so the overall N is less than the sum of NEPTUNE and CureGN sample sizes

Table S2. Factors associated with non-weight-normalized creatinine generation (mg/day, parallel to Table 3 without peds) among adults

|  | **Adult** | |
| --- | --- | --- |
|  | **Estimate (95% CI)** | **P-value** |
| Age (per 5 years) | -29.27 (-38.72, -19.87) | < 0.001 |
| Female Sex | -461.01 (-516.62, -405.32) | < 0.001 |
| Weight Status (ref: Normal Weight)  Underweight  Overweight  Obese | -62.12 (-206.15, 82.02)  146.53 (98.65, 194.69)  290.14 (236.23, 344.85) | < 0.001 |
| eGFR (per 5 mL/min/1.73m^2^) | 5.87 (2.23, 9.50) | 0.002 |
| Urine protein (per doubling) | 1.45 (-4.69, 7.64) | 0.65 |
| Non-steroid IST Use | 57.11 (20.59, 94.28) | 0.002 |
| Disease Diagnosis * Steroid Use  On Steroids (ref: MCD)  FSGS  MN  IgAN  Not on Steroids (ref: MCD)  FSGS  MN  IgAN  Steroid Use among MCD  Steroid Use among FSGS  Steroid Use among MN  Steroid Use among IgAN | 89.33 (-27.68, 206.34)  118.02 (-2.32, 238.36)  94.73 (-28.16, 217.62)  35.86 (-63.12, 134.84)  153.11 (53.93, 252.29)  168.01 (64.52, 271.50)  -33.13 (-121.72, 55.46) 20.34 (-47.28, 87.96)  -68.21 (-136.81, 0.39) -106.40 (-178.33, -34.47) | 0.07 |

Estimates are from linear mixed models with creatinine generation as the outcome and random intercepts for each study participant to account for repeated measures within individuals. The multivariable model among adult study participants includes 3445 creatinine generation measurements from 783 study participation.

Table S3. Associations between changes in creatinine generation and changes in serum creatinine between adjacent measurements (T1 and T2)

|  | **Overall** | | **Pediatric** | | **Adult** | |
| --- | --- | --- | --- | --- | --- | --- |
| **Variable** | **Estimate (95% CI)** | **P-value** | **Estimate (95% CI)** | **P-value** | **Estimate (95% CI)** | **P-value** |
| Change in Creatinine Generation  (per 1 mg/kg/day) | 0.0004 (-0.002, 0.003) | 0.78 | -0.001 (-0.01, 0.004) | 0.69 | 0.001 (-0.002, 0.004) | 0.59 |
| T1 Age (per 5 years) | -0.004 (-0.01, 0.001) | 0.11 | 0.09 (0.03, 0.15) | 0.004 | 0.001 (-0.004, 0.007) | 0.75 |
| Female Sex | -0.03 (-0.06, 0.002) | 0.07 | -0.07 (-0.19, 0.05) | 0.27 | -0.03 (-0.06, 0.01) | 0.16 |
| T1 Weight Status (ref: Normal Weight)  Underweight  Overweight  Obese | -0.05 (-0.11, 0.02)  -0.02 (-0.06, 0.03)  -0.04 (-0.08, 0.001) | 0.20 | -0.17 (-0.30, -0.02)  0.19 (0.03, 0.34)  0.01 (-0.15, 0.16) | < 0.001 | -0.01 (-0.19, 0.17)  -0.03 (-0.08, 0.01)  -0.04 (-0.08, 0.01) | 0.36 |
| Disease Diagnosis (ref: MCD)  FSGS  MN  IgAN  Non-biopsy | 0.03 (-0.02, 0.08)  -0.02 (-0.07, 0.04)  0.008 (-0.04, 0.06)  0.003 (-0.19, 0.20) | 0.24 | 0.06 (-0.09, 0.21)  0.27 (-0.01, 0.54)  -0.20 (-0.37, -0.02)  0.12 (-0.16, 0.39) | 0.02 | 0.02 (-0.04, 0.08)  -0.03 (-0.09, 0.03)  0.03 (-0.04, 0.09) | 0.05 |
| Changes in Disease Activity  (ref: not in remission at T1 nor T2)  In Remission at T1 only  In Remission at T2 only  In Remission at T1 & T2 | -0.12 (-0.16, -0.08)  -0.13 (-0.18, -0.08)  -0.13 (-0.19, -0.06) | < 0.001 | -0.11 (-0.22, -0.004)  -0.10 (-0.23, 0.02)  -0.06 (-0.20, 0.07) | 0.24 | -0.11 (-0.15, -0.07)  -0.12 (-0.18, -0.06)  -0.15 (-0.23, -0.07) | < 0.001 |
| Changes in Steroid Use  (ref: not on steroids at T1 nor T2)  On Steroids at T1  On Steroids at T2  On Steroids at T1 & T2 | -0.02 (-0.06, 0.02)  -0.05 (-0.12, 0.01)  0.03 (-0.03, 0.09) | 0.21 | 0.01 (-0.10, 0.11)  0.06 (-0.08, 0.19)  0.04 (-0.07, 0.15) | 0.79 | -0.02 (-0.07, 0.03)  -0.11 (-0.19, -0.03)  -0.03 (-0.04, 0.09) | 0.03 |
| Months between T1 and T2  (per 5 months) | 0.014 (0.01, 0.018) | < 0.001 | 0.019 (0.011, 0.029) | < 0.001 | 0.011 (0.006, 0.015) | < 0.001 |

Estimates are from linear mixed models with change in serum creatinine as the outcome and change in creatinine generation as the primary exposure, with random intercepts for each study participant to account for repeated measures within individuals. Changes are calculated for each adjacent pair of measurements, with T1 representing the time of the first measurement in the pair and T2 representing the time of the second measurement in the pair. The multivariable model among all study participants includes 2930 pairs of measurements from N=635 study participants, among pediatric study participants includes 641 pairs of measurements from N=174 study participants, and among adult study participants includes 2289 pairs of measurements from N=461 study participants.

Table S4. Associations between changes in non-weight-normalized creatinine generation (scaled to per 1 g/day, parallel to Table S2 but only for adults) and changes in serum creatinine between adjacent measurements (T1 and T2)

|  | **Adult** | |
| --- | --- | --- |
| **Variable** | **Estimate (95% CI)** | **P-value** |
| Change in Creatinine Generation  (per 1 g/day) | 0.006 (-0.03, 0.04) | 0.74 |
| T1 Age (per 5 years) | -0.004 (-0.008, 0.001) | 0.11 |
| Female Sex | -0.03 (-0.06, 0.001) | 0.07 |
| T1 Weight Status (ref: Normal Weight)  Underweight  Overweight  Obese | -0.05 (-0.11, 0.02)  -0.02 (-0.06, 0.03)  -0.04 (-0.08, 0.001) | 0.21 |
| Disease Diagnosis (ref: MCD)  FSGS  MN  IgAN | 0.03 (-0.02, 0.08)  -0.02 (-0.07, 0.04)  0.01 (-0.04, 0.06) | 0.24 |
| Changes in Disease Activity (ref: not in remission at T1 nor T2)  In Remission at T1 only  In Remission at T2 only  In Remission at T1 & T2 | -0.13 (-0.19, -0.06)  -0.13 (-0.18, -0.07)  -0.12 (-0.16, -0.08) | < 0.001 |
| Changes in Steroid Use (ref: not on steroids at T1 nor T2)  On Steroids at T1  On Steroids at T2  On Steroids at T1 & T2 | 0.03 (-0.03, 0.09)  -0.05 (-0.12, 0.01)  -0.02 (-0.06, 0.02) | 0.21 |
| Months between T1 and T2 (per 5 months) | 0.01 (0.01, 0.02) | < 0.001 |

Estimates are from linear mixed models with change in serum creatinine as the outcome and change in creatinine generation as the primary exposure, with random intercepts for each study participant to account for repeated measures within individuals. Changes are calculated for each adjacent pair of measurements, with T1 representing the time of the first measurement in the pair and T2 representing the time of the second measurement in the pair. The multivariable model among all study participants includes 2930 pairs of measurements from N=635 study participants and among adult study participants includes 2289 pairs of measurements from N=461 study participants.

Table S5. Serum creatinine-based U25 and CKD-Epi equations for estimating GFR

| **CKD-Epi (for adults aged 18 years or older)** | | | |
| --- | --- | --- | --- |
| $eGFR=142\times{\min\left( \frac{SCr}{0.7},1 \right)}^{-0.241}\times{\max\left( \frac{SCr}{0.7},1 \right)}^{-1.2}\times{0.9938}^{age}\times1.012 \text{if female}$  $eGFR=142\times{\min\left( \frac{SCr}{0.9},1 \right)}^{-0.302}\times{\max\left( \frac{SCr}{0.9},1 \right)}^{-1.2}\times{0.9938}^{age} \text{if male}$ | | | |
| **U25 (for children and adults under 25 years old)** | | |  |
|  | $eGFR=K\times\frac{height}{SCr}$ | $K=\left\{ \begin{matrix} \begin{matrix} 39.0\times{1.008}^{age-12} & \text{if male ages 1 to <12 } \\ 39.0\times{1.045}^{age-12} & \text{if male ages 12 to <18} \end{matrix} \\ \begin{matrix} 50.8 & \text{if male ages 18 to 25} \\ 36.1\times{1.008}^{age-12} & \text{if female ages 1 to <12} \end{matrix} \\ \begin{matrix} 36.1\times{1.023}^{age-12} & \text{if female ages 12 to <18} \\ 41.4 & \text{if female ages 18 to 25} \end{matrix} \end{matrix} \right.$ |  |
| Age is measured in years, height in m, SCr in mg/dL, and CysC in mg/L. U25 equations from Pierce et al. (2021); CKD-Epi equations from Inker et al. (2021). For pediatric heights, the CDC growth chart from 2000 was used to find the 50% percentile. | | |  |

**Item S1. Members of the Nephrotic Syndrome Study Network (NEPTUNE)**

**NEPTUNE Collaborating Sites**

*Atrium Health Levine Children’s Hospital, Charlotte, SC*: Susan Massengill^*^, Layla Lo^#^

*Cleveland Clinic, Cleveland, OH*: Katherine Dell^*^, John O’Toole^*^, John Sedor^**^, Victoria Grange^#^

*Children’s Hospital, Los Angeles, CA*: Ian Macumber^*^, Alyssa Parry^#^

*Children’s Mercy Hospital, Kansas City, MO*: Tarak Srivastava^*^, Kelsey Markus^#^

*Cohen Children’s Hospital, New Hyde Park, NY*: Christine Sethna^*^, Suzanne Vento^#^

*Columbia University, New York, NY:* Pietro Canetta^*^

*Duke University Medical Center, Durham, NC:* Opeyemi Olabisi^*^, Rasheed Gbadegesin^**^, Maurice Smith^#^

*Emory University, Atlanta, GA:* Laurence Greenbaum^*^, Chia-shi Wang^*^, Emily Yun^#^

*The Lundquist Institute, Torrance, CA:* Sharon Adler^*^, Janine LaPage^#^

*John H Stroger Cook County Hospital, Chicago, IL:* Amatur Amarah^*^

*Johns Hopkins Medicine, Baltimore, MD:* Meredith Atkinson^*^, Ryan Hutson^#^

*Mayo Clinic, Rochester, MN:* John Lieske, Marie Hogan, Fernando Fervenza

*Medical University of South Carolina, Charleston, SC:* David Selewski^*^, Cheryl Alston^#^

*Montefiore Medical Center, Bronx, NY:* Kim Reidy^*^, Michael Ross^*^, Frederick Kaskel^**^, Patricia Flynn^#^

*New York University Medical Center, New York, NY:* Laura Malaga-Dieguez^*^, Olga Zhdanova^**^, Laura Jane Pehrson^#^, Melanie Miranda^#^

*The Ohio State University College of Medicine, Columbus, OH*: Salem Almaani^*^, Laci Roberts^#^

*Stanford University, Stanford, CA:* Richard Lafayette^*^, Shiktij Dave^#^

*Temple University, Philadelphia, PA:* Iris Lee^**^

*Texas Children’s Hospital at Baylor College of Medicine, Houston, TX*: Shweta Shah^*^, Sadaf Batla^#^ ^#^

*University Health Network Toronto:* Heather Reich^*^, Michelle Hladunewich^**^, Paul Ling^#^, Martin Romano^#^

*University of California at San Francisco, San Francisco, CA*: Paul Brakeman^*^, Daniel Schrader

*University of Colorado Anschutz Medical Campus, Aurora, CO*: James Dylewski^*^ Nathan Rogers^#^

*University of Kansas Medical Center, Kansas City, KS*: Ellen McCarthy^*^, Catherine Creed^#^

*University of Miami, Miami, FL:* Alessia Fornoni^*^, Miguel Bandes^#^

*University of Michigan, Ann Arbor, MI:* Matthias Kretzler^*^, Laura Mariani^*^, Zubin Modi^*^, A Williams^#^, Roxy Ni^#^

*University of Minnesota, Minneapolis, MN:* Patrick Nachman^*^, Michelle Rheault^*^, Amy Hanson^#^, Nicolas Rauwolf^#^

*University of North Carolina, Chapel Hill, NC:* Vimal Derebail^*^, Keisha Gibson^*^, Anne Froment^#^, Mary Mac McGown Collie^#^

*University of Pennsylvania, Philadelphia, PA:* Lawrence Holzman^*^, Kevin Meyers^**^, Krishna Kallem^#^, Aliya Edwards^#^

*University of Texas San Antonio, San Antonio, TX*: Samin Sharma^**^

*University of Texas Southwestern, Dallas, TX:* Elizabeth Roehm^*^, Kamalanathan Sambandam^**^, Elizabeth Brown^**^, Jamie Hellwege

*University of Washington, Seattle, WA:* Ashley Jefferson^*^, Sangeeta Hingorani^**^, Katherine Tuttle^**§^, Linda Manahan ^#^, Emily Pao^#^, Kelli Kuykendall^§^

*Wake Forest University Baptist Health, Winston-Salem, NC:* Jen Jar Lin^**^

*Washington University in St. Louis, St. Louis, MO*: Vikas Dharnidharka^*^

**Data Analysis and Coordinating Center:** *University of Michigan:* Matthias Kretzler^*^, Brenda Gillespie^**^, Laura Mariani^**^, Zubin Modi^**^, Eloise Salmon^**^, Howard Trachtman^**^, Tina Mainieri, Gabrielle Alter, Michael Arbit, Hailey Desmond, Sean Eddy, Damian Fermin, Wenjun Ju, Maria Larkina, Chrysta Lienczewski, Rebecca Scherr, Jonathan Troost, Amanda Williams, Yan Zhai; *Arbor Collaborative for Health:* Colleen Kincaid, Shengqian Li, Shannon Li; *Cleveland Clinic:* Crystal Gadegbeku^**^, *Duke University:* Laura Barisoni^**^; John Sedor^**^, *Harvard University:* Matthew G Sampson^**^; *Northwestern University:* Abigail Smith^**^; *University of Pennsylvania:* Lawrence Holzman^**^, Jarcy Zee^**^

**Digital Pathology Committee:** Carmen Avila-Casado *(University Health Network)*, Serena Bagnasco *(Johns Hopkins University)*, Lihong Bu *(Mayo Clinic)*, Shelley Caltharp *(Emory University)*, Clarissa Cassol *(Arkana)*, Dawit Demeke *(University of Michigan)*, Brenda Gillespie *(University of Michigan)*, Jared Hassler *(Temple University)*, Leal Herlitz *(Cleveland Clinic)*, Stephen Hewitt *(National Cancer Institute)*, Jeff Hodgin *(University of Michigan)*, Danni Holanda *(Arkana)*, Neeraja Kambham *(Stanford University)*, Kevin Lemley, Laura Mariani *(University of Michigan)*, Nidia Messias *(Washington University)*, Alexei Mikhailov *(Wake Forest)*, Vanessa Moreno *(University of North Carolina)*, Behzad Najafian *(University of Washington)*, Matthew Palmer *(University of Pennsylvania)*, Avi Rosenberg *(Johns Hopkins University)*, Virginie Royal *(University of Montreal)*, Miroslav Sekulik *(Columbia University)*, Barry Stokes *(Columbia University)*, David Thomas *(Duke University)*, Ming Wu *(University of New York)*, Michifumi Yamashita *(Cedar Sinai)*, Hong Yin *(Emory University)*, Jarcy Zee *(University of Pennsylvania)*, Yiqin Zuo *(University of Miami)*. Co-Chairs: Laura Barisoni *(Duke University)*, Cynthia Nast *(Cedar Sinai)*.

**Item S2. Cure Glomerulonephropathy (CureGN) Collaborators**

The CureGN Consortium members listed below, from within the four Participating Clinical Center networks and Data Coordinating Center, are acknowledged by the authors as Collaborators.

**CureGN Principal Investigators; *CureGN Site Principal Investigators; ^#^CureGN Lead Coordinators.

**CureGN Participating Clinical Centers (PCC) through Columbia University:**

*Columbia University, New York, NY, US*: Wooin Ahn, Gerald Appel, Paul Appelbaum, Revekka Babayev, Andrew Bomback, Pietro Canetta, Brenda Chan, Vivette Denise D'Agati, Samitri Dogra, Hilda Fernandez, Ali Gharavi^**^, William Hines, Syed Ali Husain, Namrata Jain, Krzysztof Kiryluk, Fangming Lin, Maddalena Marasa^#^, Glen Markowitz, Hila Milo Rasouly, Sumit Mohan, Nicola Mongera, Jordan Nestor, Thomas Nickolas, Jai Radhakrishnan, Maya Rao, Simone Sanna-Cherchi, Shayan Shirazian, Michael Barry Stokes, Natalie Uy, Anthony Valeri, Natalie Vena

*University of Warsaw, Warszawa, Poland:* Bartosz Foroncewicz, Barbara Moszczuk, Krzysztof Mucha*, Agnieszka Perkowska-Ptasińska

*Gaslini Children’s Hospital, Genoa, Italy:* Gian Marco Ghiggeri*, Francesca Lugani

**CureGN Participating Clinical Centers (PCC) through the Pediatric Nephrology Research Consortium:**

*Arkana Laboratories, Little Rock, AR, USA*: Josephine Ambruzs, Helen Liapis

*Children’s Hospital of Michigan, Detroit, MI, USA*: Rossana Baracco, Amrish Jain*

*Children’s Hospital of New Orleans/ LSU Health, New Orleans, LA, USA*: Isa Ashoor, Diego Aviles*

*Children’s Mercy Hospital, Kansas City, MO, USA*: Tarak Srivastava*

*Children’s National Medical Center, Washington DC, USA*: Sun-Young Ahn*

*Cincinnati Children’s Hospital Cincinnati, OH, USA*: Prasad Devarajan, Elif Erkan*, Donna Claes, Hillarey Stone

*Connecticut Children’s Medical Center, Hartford, CT, USA*: Sherene Mason*

*Duke Children’s Hospital Medical Center, Durham, NC, USA*: Rasheed Gbadegesin*

*East Carolina University Brody School of Medicine, Greenville, NC, USA*: Liliana Gomez-Mendez*

*Emory University, Atlanta, GA, USA*: Larry Greenbaum**, Chia-shi Wang, Hong (Julie) Yin

*Helen DeVos Children’s Hospital, Grand Rapids, MI, USA*: Yi Cai*, Goebel Jens, Julia Steinke

*Levine Children’s Hospital/Atrium Health, Charlotte, NC, USA*: Donald Weaver*

*Lurie Children’s Hospital, Chicago IL, USA*: Jerome Lane*

*Mayo Clinic, Rochester, MN, USA*: Carl Cramer*

*Medical College of Wisconsin, Milwaukee, WI, USA*: Cindy Pan, Neil Paloian, Rajasree Sreedharan*

*Medical University of South Carolina, Charleston SC, USA*: David Selewski, Katherine Twombley*

*Nationwide Children’s Hospital, Columbus, OH, USA*: Corinna Bowers^#^, Mary Dreher^#^ Mahmoud Kallash*, John Mahan, Samantha Sharpe^#^, William Smoyer**

*Oregon Health and Science University, Portland, OR, USA*: Amira Al-Uzri*, Sandra Iragorri

*Riley Children’s Hospital, Indianapolis, IN, USA*: Myda Khalid*

*Cardinal Glennon Children’s Medical Center/ St. Louis University, St. Louis, MO, USA*: Craig Belsha*

*Texas Children’s Hospital, Houston, TX, USA*: Joseph Alge*, Michael Braun, AC Gomez, Scott Wenderfer*

*Texas Tech Health Sciences Center, Amarillo, TX, USA*: Tetyana Vasylyeva*

*Children’s of Alabama, University of Alabama, Birmingham, AL, USA*: Daniel Feig*

*University of Colorado Children’s Hospital, Colorado, Aurora, CO, USA*: Gabriel Cara Fuentes, Melisha Hannah*

*University of Iowa Children’s Hospital, Iowa City, IA, USA*: Carla Nester*

*University of Kentucky, Lexington, KY, USA*: Aftab Chishti*

*University of Louisville, Louisville, KY, USA*: Jon Klein^**^

*Holtz Medical Center, University of Miami, Miami, FL, USA*: Chryso Katsoufis, Wacharee Seeherunvong*

*University of Minnesota Children’s Hospital, Minneapolis, MN, USA*: Michelle Rheault*

*University of New Mexico Health Sciences Center, Albuquerque, NM, USA*: Craig Wong*

*University of Oklahoma Health Sciences Center, Oklahoma City, OK, USA*: Nisha Mathews*

*University of Virginia, Charlottesville, VA, USA*: John Barcia*, Agnes Swiatecka-Urban

*University of Wisconsin, Madison, WI, USA*: Sharon Bartosh*

*Vanderbilt Children’s Hospital, Nashville TN, USA*: Tracy Hunley*

*Washington University in St. Louis, St. Louis, MO, USA*: Vikas Dharnidharka*, Joseph, Gaut

**CureGN Participating Clinical Centers (PCC) through the University of North Carolina:**

*Hôpital Maisonneuve-Rosemont, Montreal, Canada*: Louis-Philippe Laurin*, Virginie Royal

*Medical University of South Carolina, Charleston, SC, USA*: Anand Achanti, Milos Budisavljevic*, Sally Self

*Northwestern University, Chicago, IL, USA*: Cybele Ghossein, Yonatan Peleg, Shikha Wadhwani*

*Ohio State University, Columbus, OH, USA*: Salem Almaani, Isabelle Ayoub, Tibor Nadasdy, Samir, Parikh, Brad Rovin*

*University of Chicago, Chicago, IL, USA*: Anthony Chang

*University of Alabama at Birmingham, Birmingham, AL, USA*: Huma Fatima, Bruce Julian, Jan Novak, Matthew Renfrow, Dana Rizk*

*University of North Carolina Kidney Center, Chapel Hill, NC, USA*: Dhruti Chen, Vimal Derebail, Ronald Falk**, Keisha Gibson, Dorey Glenn, Susan Hogan, Koyal Jain, J. Charles Jennette, Amy Mottl*, Caroline Poulton^#^, Manish Kanti Saha

*Vanderbilt University, Nashville, TN, USA*: Agnes Fogo, Neil Sanghani*

*Virginia Commonwealth University, Richmond, VA, USA*: Jason Kidd*, Selvaraj Muthusamy

**CureGN Participating Clinical Centers (PCC) through the University of Pennsylvania:**

*MetroHealth Medical Center/Case Western Reserve University, Cleveland, OH, USA*: Jeffrey Schelling*

*Cedars-Sinai Health System, Los Angeles, CA, USA*: Jean Hou

*Children’s Hospital of LA, Los Angeles, CA, USA*: Kevin Lemley*, Warren Mika, Pierre Russo

*Children’s Hospital of Philadelphia, Philadelphia, PA, USA*: Michelle Denburg, Amy Kogon, Kevin Meyers*, Madhura Pradhan

*Cleveland Clinic, Cleveland, OH, CA*: Raed Bou Matar*, John O'Toole*, John Sedor*

Cohen Children’s Medical Center, New Hyde Park, NY, USA: Christine Sethna*, Suzanne Vento ^#^

*Johns Hopkins University, Baltimore, MD, USA*: Mohamed Atta, Serena Bagnasco, Alicia Neu, John Sperati*

*Lundquist Institute at Harbor-UCLA Medical Center, Torrance, CA, USA*: Sharon Adler*, Tiane Dai, Ram Dukkipati

*Mayo Clinic, Rochester, MN, USA*: Fernando Fervenza*, Sanjeev Sethi

*Montefiore Medical Center, The Bronx, New York, NY, USA*: Frederick Kaskel, Kaye Brathwaite, Kimberly Reidy*

*New York University, New York, NY, USA*: Joseph Weisstuch, Ming Wu, Olga Zhdanova

*NIDDK, Bethesda, MD, USA*: Jurgen Heymann, Jeffrey Kopp*, Meryl Waldman, Cheryl Winkler

*Spokane Providence Medical Center, Spokane, WA, USA*: Katherine Tuttle*

*Stanford University, Palo Alto, CA, USA*: Jill Krissberg, Richard Lafayette*, Kamal Fahmeedah, Elizabeth Talley

*Sunnybrook Health Sciences Centre, Toronto, Canada*: Michelle Hladunewich*

*The Hospital for Sick Children, Toronto, Canada*: Rulan Parekh*

*University Health Network, Toronto, Canada*: Carmen Avila-Casado, Daniel Cattran*, Reich Heather, Philip Boll

*University of Miami, Miami, FL, USA*: Yelena Drexler, Alessia Fornoni*

*University of Michigan, Ann Arbor, MI, USA*: Brooke Blazius*, Jeffrey Hodgin, Andrea Oliverio

*University of Pennsylvania, Philadelphia, PA, USA*: Jon Hogan, Lawrence Holzman**, Matthew Palmer, Gaia Coppock

*University of Pittsburgh School of Medicine, Pittsburgh, PA, USA*: Blaise Abromovitz*, Michael Mortiz*

*University of Washington, Seattle, WA, USA*: Charles Alpers, J. Ashley Jefferson*

*UT Southwestern, Dallas, TX, USA*: Elizabeth Brown, Kamal Sambandam*, Bethany Roehm

**Data Coordinating Center (DCC):**

*Arbor Research Collaborative for Health, Ann Arbor, MI, USA*: John Graff, Abigail Smith

*Cedar Sinai Medical Center, Los Angeles, CA, USA*: Cynthia Nast

*Duke University, Durham, NC, USA*: Laura Barisoni

*University of Michigan, Ann Arbor, MI, USA*: Brenda Gillespie**, Bruce Robinson**, Matthias Kretzler, Laura Mariani**

**Steering Committee Chair:** Lisa M. Guay-Woodford, Children’s Hospital of Pennsylvania, Philadelphia, PA, USA
